# Certified service dogs – A cost-effectiveness analysis appraisal

**DOI:** 10.1371/journal.pone.0219911

**Published:** 2019-09-12

**Authors:** Martina Lundqvist, Jenny Alwin, Lars-Åke Levin

**Affiliations:** Department of Medical and Health Sciences, Linkoping University, Linköping, Sweden; Massachusetts General Hospital, UNITED STATES

## Abstract

**Introduction:**

Individuals with functional impairments or chronic diseases are often in need of assistance in their daily lives. For these individuals it is essential to find novel, cost-effective solutions to meet their needs. Service dogs are dogs that are specially trained to assist individuals with functional impairments and may be able to improve these individuals’ quality of life at a reasonable cost, i.e. be cost effective. Cost-effectiveness analyses are used to illustrate the cost of an intervention in relation to its effects and provide important input to decision-makers when setting priorities.

**Aim:**

The aim of this study is to assess the cost effectiveness of a certified physical service dog and a diabetes alert dog compared to a regular companion dog.

**Method:**

Costs, life years and quality-adjusted life years were estimated over a 10-year time horizon using a decision-analytic model built upon evidence from the”service and hearing dog project”. The primary outcome was the incremental cost-effectiveness ratio expressed as cost per gained quality-adjusted life year. The analysis was conducted from a societal perspective. Costs and effects were discounted with 3% per annum and reported in USD.

**Results:**

Compared to a regular companion dog, a physical service dog is cost saving [-6,000 USD] and gives the dog owner more quality-adjusted life years [0.28]. The diabetes alert dog is also cost effective in comparison with a regular companion dog [-4,500 USD, 0.06 QALYs].

**Conclusion:**

This study indicates that a certified service dog is cost saving in comparison with a regular companion dog for individuals with functional impairments or chronic diseases. The uncertainty of the analysis implies that further studies are needed in order to confirm these results. Nevertheless, physical service dogs and diabetes alert dogs show potential to be a valuable support and decision analytic models are useful tools to provide this information.

## Introduction

Individuals with functional impairments or chronic diseases such as intractable diabetes or epilepsy are often in need of a lot of health and social care services [1]. In addition, they often need help from informal caregivers in order to be able to function in their everyday life. Hence, it is essential to find means and solutions that meet the needs of these individuals and improve their quality of life. One solution to this might be the use of certified assistance dogs.

“Assistance dog” is an umbrella term that includes guide dogs, hearing dogs and service dogs, where service dogs can be divided into subgroups of physical service dogs, diabetes alert dogs and seizure alert dogs, etc. [2]. Physical service dogs are specially trained to assist individuals with functional impairments and to help their owner in everyday life with, for example, getting dressed, picking up dropped items, opening and closing doors, and in emergency situations, or if the owner needs help, attracting another person’s attention. Diabetes and seizure alert dogs can alert their owners of low or high blood sugar levels or imminent seizures, respectively. An assistance dog can also be trained to be a hearing dog. A hearing dog serves individuals who are deaf or have a hearing impairment. Their primary task is to alert their owners of different sounds e.g. door bells, fire alarms, a phone ringing, etc. [3, 4].

In a previous study measuring the short-term effects of a certified service or hearing dog, it was shown that certified dogs tend to improve their owners’ health-related quality of life (HRQoL) [5]. Consequently, there is reason to believe that the skills of the dog and the collaboration and attachment between the owner and the dog can develop over time and have positive long-term effects.

Applying economic evaluation methods makes it possible to examine the long-term effects of an assistance dog, both in terms of resource use affected by the dog and the health outcomes of having an assistance dog. Cost-effectiveness analyses are also used to inform decision-makers. They are a tool to systematically weigh costs against health effects and to compare relevant alternatives, which is necessary in a health and social care system with scarce resources and endless needs. In order to estimate the long-term costs and effects in health-economic analyses, economic decision models are used. These models make it possible to use data from clinical trials and other sources and to estimate what the results will be over longer periods of time. Since models are associated with uncertainty, sensitivity analyses are conducted to examine the effects of individual data input on the results. In the present study we set out to make a first attempt at using health-economic decision modeling to provide information on the cost effectiveness of certified dogs. Findings from our previous study showed that the service and hearing dog owners where a heterogonous group [5]. Therefore this study will focus on the physical service dog and the diabetes alert dog owners only.

The aim of this study was to assess the cost effectiveness of a certified physical service dog and hearing diabetes alert dog in comparison to a regular companion dog.

## Methods

### Overview of the analytical approach

The cost-effectiveness analysis of certified dogs for persons with functional impairments was based on a decision-analytic Markov model with a 10-year time horizon. A Markov model is a stochastic model that describes a process, in this case the life of a dog owner with functional impairments, over a finite set of outcomes, usually called states. In this model the state transitions happens with one year fixed cycles. At the end of each cycle, the patients move from one health state to another, or remain in the same state, according to set probabilities for each transition. This implies that future transitions in the model happen independently of previous chains of events.

Our model estimated the marginal cost and the effects of adding certified dogs to hypothetical persons who matched the population of the individuals in the Swedish “service and hearing dog project” 2009–2014 [5]. The costs were calculated in Swedish kronor (SEK) and converted to 2017 US dollars (USD) using the exchange rate of 1 USD = 8.538 SEK (year 2017 mean exchange rate). The effect was expressed as quality-adjusted life years (QALYs). The QALY is a composite measure combining information about HRQoL and length of life. The primary outcome of the model was the incremental cost-effectiveness ratio (ICER) expressed as cost per QALY gained:

$$ICER=\frac{{Costs}_{Certified\;dog}-{Costs}_{Companion\;dog}}{{QALYs}_{Certified\;dog}-{QALYs}_{Companion\;dog}}$$

Cost and QALYs were discounted by 3 percent annually. The base-case analysis, i.e. the analysis with the most likely set of assumption and input values, was conducted from a societal perspective, meaning that all relevant costs affected by the intervention were included.

### The service and hearing dog project

The main data source for the analysis was the “service and hearing dog project” reported in detail in previous publication [5]. In short, the “service and hearing dog project” was a longitudinal interventional study with a pre-post design where dog owners and their companion dogs were included. The intervention was to train the dogs to become certified service or hearing dogs. All participants gave written informed consent to participate in the study. The study collected data on resource use and HRQoL. Baseline data was collected prior to the start of the training and follow-up data was collected three months after the dog was certified. The baseline data in the model represents the resource use and health effects of having a regular pet dog while the follow-up data corresponds to the resource use and health effects of having a certified dog. Fifty-five owners managed to certify their dogs, including 30 physical service dogs, 20 diabetes alert dogs, 3 hearing dogs and 2 seizure alert dogs. In this article, the analysis will be based on data from the physical service dog owners and the diabetes alert dog owners.

The study was approved by the regional ethics vetting board Linköping University (No: 157–09) and retrospectively registered in clinicaltrial.gov, NCT03270592, September 2017.

### The decision-analytic Markov model

**Fig 1:**
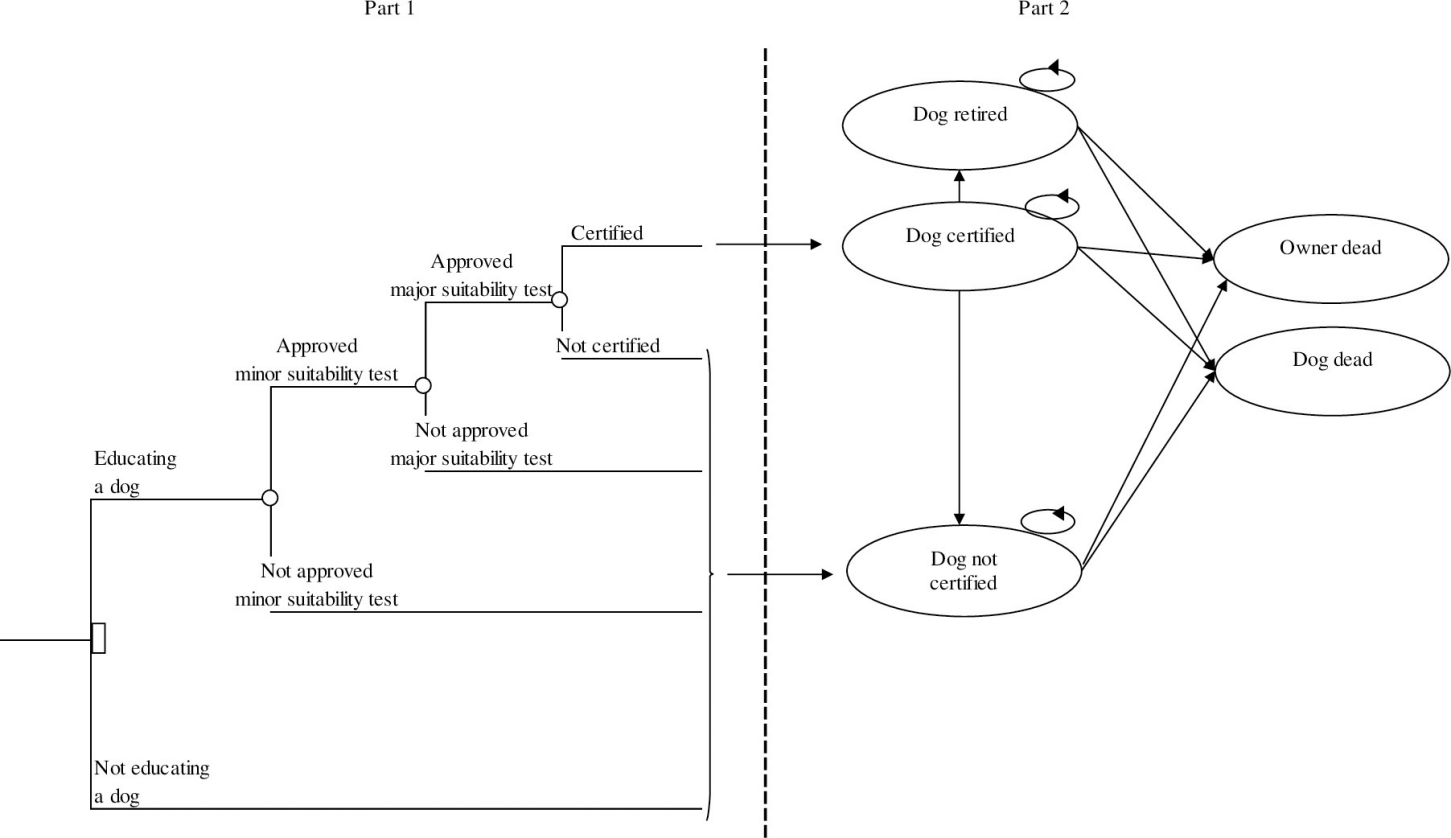
Structure of the decision-analytic Markov model. The decision of training or not training a certified dog is shown in part 1 of the figure. Part 2 describes how owners training a certified dog can experience that the dog is retired or loses its certification and that the human as well as the dog faces an annual risk of dying. Owners not training a dog can move from the ‘Dog not certified’ state to ‘Owner dead’ or ‘Dog dead’ state.

The model structure is illustrated in Fig 1. Part 1, the one-year decision tree model, shows that owners who wish to train their dog to be certified must undergo and pass initial tests (one minor and one major suitability test) to determine if the dog and owner, as a unit, are suitable for the training. After the training, the owner and dog have to pass a final exam in order to gain certification. Owners who do not pass the minor and major suitability tests, the final exam, or who do not want to train a dog move directly to part 2 in the model (Fig 1). The probabilities for passing the minor and major suitability tests and for passing the examination were based on data from the “service and hearing dog project”, Table 1. The probabilities for transferring between the states were based on assumptions determined in consultation with the Swedish Association of Service Dogs, Table 1. During each year, owners of a certified dog can move from having a certified dog to the ‘Dog retired’ state or if they do not pass the annual certification maintenance test they can move to the ‘Dog not certified’ state (part 2, Fig 1). The owners as well as the dogs also face an annual risk of death, which shifts them to the ‘Owner dead’ or ‘Dog dead’ state. Owners of a regular companion dog can move from the ‘Dog not certified’ state to the ‘Owner dead’ state or the ‘Dog dead’ state. After the initial decision made in model part 1, the Markov model runs for 9 years, based on the time a dog is expected to be used as a certified dog.

**Table 1:** Parameters used in the model for physical service dog owners.

Model parameter	Regular companion dog (alfa, beta)	Certified dog (alfa, beta)	Retired dog/dog that lost certification status (alfa, beta)
Annual costs*/owner (USD)			
**Health care**			
Hospitalization	617	378	497
Emergency care	1,500	833	1,167
Ambulance	101	-	50
Visit to physicians (hospital)	528	793	661
Visit to physicians (health center)	925	691	808
Home visit physicians	69	-	34
Visit to nurse	338	987	663
Home visit nurse	507	507	507
Visit to physiotherapist	1,791	1,360	1,576
Home visit physiotherapist	-	100	50
Visit to occupational therapist	391	287	339
Home visit occupational therapist	417	313	365
Visit to other caregiver	1,743	1,282	1,513
Home visit other caregiver	-	-	-
*Total*	8,928 (27, 704)	7,530 (23, 703)	8,229 (25, 702)
**Municipal services**			
Home-help services	648	626	637
Personal assistants	40,042	42,879	41,461
Escort/accompanying person	811	1,060	935
Transportation service	2,449	2,357	2,403
Other service	638	708	673
*Total*	44,588 (10, 9596)	47,630 (9, 11232)	46,109 (9, 10408)
**Informal care**	4,768 (20, 501)	3,503 (26, 283)	4,135 (23, 391)
**Sick leave**	66,901 (228, 627)	63,859 (147, 926)	65,380 (182, 768)
**Dog costs year 1**			
Purchase dog	1,151 (308, 32)	1,151 (308, 32)	1,151 (308, 32)
Annual costs**	1,332 (92, 10)	1,332 (92, 10)	1,332 (92, 10)
Suitability tests	-	193	-
*Total*	2,482	2,676	2,482
**Dog costs year 2**			
Annual costs**	1,332 (92, 10)	1,332 (92, 10)	1,332 (92, 10)
Dog training	-	7,746	-
Cost for capes	-	211	-
*Total*	1,332	9,288	1,332
**Dog costs following years**			
Annual costs**	1,332 (92, 10)	1,332 (92, 10)	1,332 (92, 10)
Annual certification maintenance test	-	88	-
Annual health declaration	-	59	-
*Total*	1,332	1,478	1,332
**QALY weights**‡	0.226 (14, 39)	0.351 (29, 53)	0.309

*All costs were assumed to be gamma distributed.

** Annual costs includes costs for food, insurance and veterinary costs QALY = Quality Adjusted Life Years.

‡All QALY weights were assumed to be beta distributed.

### Data

#### Costs

Cost estimations for owners of a regular companion dog were based on the baseline data from the “service and hearing dog project”. Costs estimations for owners of a certified physical service dog and a certified diabetes alert dog were based on the follow-up data. For owners of a dog that lost its certification status or if the dog was retired, we assumed a mean cost in between the cost for a regular companion dog and the cost for a certified dog, Tables 1 and 2. If the dog died, costs were estimated based on the baseline data. The costs for different states were applied as long as the owners remained in the states, respectively.

**Table 2:** Parameters used in the model for diabetes alert dog owners.

Model parameter	Regular companion dog (alfa, beta)	Certified dog (alfa, beta)	Retired dog/dog that lost certification status (alfa, beta)
Annual costs*/owner (USD)			
**Health care**			
Hospitalization	1,770	3,017	2,393
Emergency care	3,250	750	2,000
Ambulance	831	151	491
Visit to physicians (hospital)	1,633	1,427	1,530
Visit to physicians (health center)	309	566	437
Home visit physicians	-	-	-
Visit to nurse	994	1,015	1,004
Home visit nurse	-	-	-
Visit to physiotherapist	75	1,567	821
Home visit physiotherapist	100	-	50
Visit to occupational therapist	-	1,055	527
Home visit occupational therapist	78	-	39
Visit to other caregiver	437	454	446
Home visit other caregiver	-	-	-
*Total*	9,475 (5, 3752)	10,001 (12, 1805)	9,738 (8, 2677)
**Municipal services**			
Home-help services	-	-	-
Personal assistants	3,474	3,040	3,257
Escort/accompanying person	-	-	-
Transportation service	25	38	32
Other service	28	17	23
*Total*	3,528 (1, 7292)	3,095 (1, 6417)	3,312 (1, 6855)
**Informal care**	2,414 (12, 432)	1,168 (13, 198)	1,791 (12, 315)
**Sick leave**	28,900 (15, 4178)	27,829 (12, 4822)	28,364 (13, 4491)
**Dog costs year 1**			
Purchase dog	1,151 (308, 32)	1,151 (308, 32)	1,151 (308, 32)
Annual costs**	1,332 (92, 10)	1,332 (92, 10)	1,332 (92, 10)
Suitability tests	-	193	-
*Total*	2,482	2,676	2,482
**Dog costs year 2**			
Annual costs**	1,332 (92, 10)	1,332 (92, 10)	1,332 (92, 10)
Dog training	-	7,746	-
Cost for capes	-	211	-
*Total*	1,332	9,288	1,332
**Dog costs following years**			
Annual costs**	1,332 (92, 10)	1,332 (92, 10)	1,332 (92, 10)
Annual certification maintenance test	-	88	-
Annual health declaration	-	59	-
*Total*	1,332	1,478	1,332
**QALY weights**‡	0.656 (36, 19)	0.674 (24, 12)	0.665

*All costs were assumed to be gamma distributed.

** Annual costs includes costs for food, insurance and veterinary costs QALY = Quality Adjusted Life Years.

‡All QALY weights were assumed to be beta distributed.

Information on health-care utilization was collected by asking the participants, in a telephone interview, how much health care they had used during the past three months and whether or not the health care was related to the reason for educating a certified dog. Data on related health-care utilization were multiplied with unit costs mainly obtained from two different regional price lists (Pricing and payment for healthcare in the Southeastern region of Sweden 2017 and Pricing and payment for healthcare in the Southern region of Sweden 2017) [6, 7]. Cost of informal care was valued based on loss of leisure time, where one hour was valued as 35 percent of average gross wage [8, 9]. Productivity loss due to sick leave was valued as gross wage including payroll taxes [10]. The dog costs were obtained from the patient survey and information from the Swedish Association of Service Dogs. To estimate the annual mean costs in Tables 1 and 2, the quarterly costs were multiplied by four. Unit costs used are presented in S1 Appendix.

#### Quality adjusted life years

To estimate the QALY weights for the states in the model, HRQoL data for the mean participant in the “service and hearing dog project” were used. The HRQoL data for the regular companion dog state was based on the baseline data and the HRQoL data for having a certified dog was obtained from the follow-up. If the dog lost its certification or retired, we assumed that the owner obtained a QALY weight in between the estimate for owners of a regular companion dog and owners of a certified dog. If the dog died we assumed the owner obtained the baseline QALY weight. The EQ-5D instrument was used and converted to QALY weights using the UK value set ranging from -0.594 to 1 [11]. The different QALY weights used in the model for physical service dog owners and for diabetes alert dog owners are presented in in Tables 1 and 2 respectively.

#### Mortality

The model used age-based standard mortality rates for the general population in Sweden [12]. In addition, the model used mortality rates for dogs from a study conducted by Egenvall et al. 2005 [13].

#### Analysis

The analyses were conducted from a societal perspective, meaning that all relevant costs affected by the intervention were included in the analyses, even costs for productivity loss and informal care. The starting age of the cohort (44 years) was based on the mean age of the owners in the “service and hearing dog project”. The starting age of the dog was set to two years. The dog was assumed to retire when it reached 10 years of age.

A probabilistic sensitivity analysis was employed to evaluate the uncertainty in the incremental cost effectiveness results due to sampling uncertainty in estimated values of input parameter [14]. This analysis, conducted with simulation technique (10,000 simulations) and presented in the cost-effectiveness plane, indicates the uncertainty guiding the decision to implement or not implement the strategy. The probability for a certified dog being cost effective at different threshold values (i.e. different maximum values for an acceptable cost per gained QALY) was reported using cost-effectiveness acceptability curves [15].

One-way sensitivity analyses were carried out to investigate simplifications and assumption that were not associated with statistical uncertainty. Sensitivity analyses were carried out for different discount rates, the life span of the dog, the dog’s retirement age, the cost of purchasing a fully trained dog, the costs and QALY estimates of having a retired dog, the inclusion of health-care utilization unrelated to educating a certified dog (i.e. all health-care utilization), excluding productivity losses and changing the perspective of the analysis to a narrower health-care perspective. Values used for the deterministic sensitivity analysis are presented in the result section.

All statistical analyses were performed in SPSS version 23.0 [16]. The decision-analytic model was programmed and analyzed in Microsoft Excel (Microsoft Corporation, Redmond, Washington DC, USA).

## Results

Compared to having a regular companion dog, the owners of both physical service and diabetes alert dogs over a 10-year horizon used less resources of health care, informal care and also showed reduced productivity loss. Owners of a diabetes alert dog also used less resources of municipal services. The cost of having a certified dog was higher than for the regular companion dog, mainly because of the cost of training the dog, Table 3.

**Table 3:** Costs divided into different categories for certified dog owners and regular companion dog owners. The costs are estimated over a 10-year time horizon.

	Certified dog	Regular companion dog	Certified dog-Regular companion dog
*Physical service dog*			
*Costs (USD)*			
Health-care costs	72,804	84,394	-11,590
Municipal services	431,484	421,479	10,005
Informal care	40,909	45,068	-4,159
Productivity loss	622,396	632,399	-10,004
Dog	16,536	13,740	2,796
**Total costs**	1,184,128	1,197,080	-12,952
*Diabetes alert dog*			
*Costs (USD)*			
Health-care costs	76,448	89,566	-13,118
Municipal services	31,926	33,348	-1,422
Informal care	18,720	22,818	-4,098
Productivity loss	269,658	273,181	-3,523
Dog	16,536	13,740	2,796
**Total costs**	413,288	432,653	-19,365

The result of the cost-effectiveness analysis showed that a physical service dog compared to a regular companion dog was a dominant alternative, i.e. achieved both lower costs [-6,000 USD] and a gain in QALYs [0.28], see Table 4. Similar result was achieved for the diabetes alert dogs [-4,500 USD, 0.06 QALYs]. The reduced costs are explained by the overall reduction in health-care utilization. There was no difference in mortality between the alternatives, the QALY gain is entirely explained by the improved quality of life.

**Table 4:** Cost effectiveness of a physical service dog compared to a regular companion dog and cost effectiveness of a diabetes alert dog compared to a regular companion dog.

	Costs (USD)	ΔCost (USD)	QALY	ΔQALY	Cost per QALY gained
*Physical service dog*					
Certified	1,191,121	-5,959	2.79	0.28	Dominant
Regular companion	1,197,080		2.51		
*Diabetes alert dog*					
Certified	428,137	-4,516	6.26	0.06	Dominant
Regular companion	432,653		6.20		
QALY = Quality Adjusted Life Years					

### Sensitivity analysis

**Fig 2:**
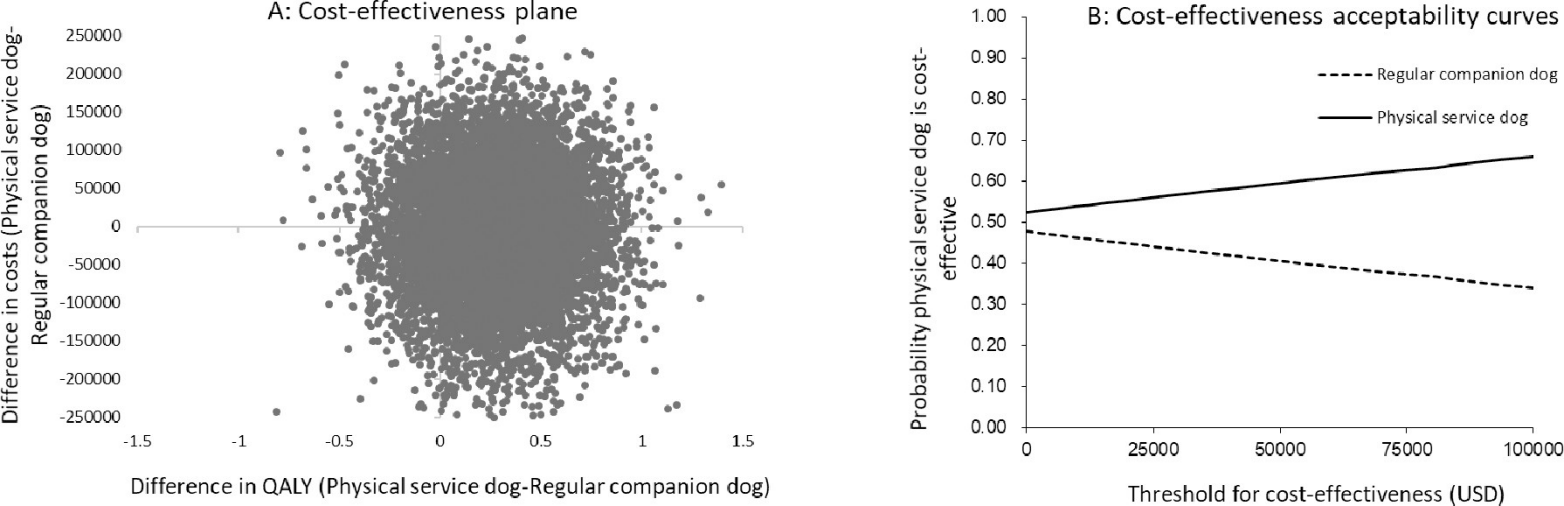
Result of probabilistic analysis for physical service dog owners. Panel A: Cost-effectiveness plane based on 10,000 iterations illustrating the distribution of the ICERs. Panel B: Cost-effectiveness acceptability curves showing the probability that a certified physical service dog is cost effective at different thresholds for cost effectiveness.

The result from the probabilistic sensitivity analysis for physical service dogs is illustrated in the cost-effective plane in Fig 2 (panel A). The distribution reveals that the result was uncertain, since the simulation estimates were spread over all the quadrants. However, the joint distribution of incremental costs and QALYs reveals that a certified service dog is associated with a decrease in costs in 52 percent of the simulations and a gain in QALYs in 87 percent of the simulations. The probability for physical service dog being cost effective at different threshold values is shown in Fig 2 (panel B). The curves reveal that the probability for a physical service dog being cost effective increases as the threshold increase.

**Fig 3:**
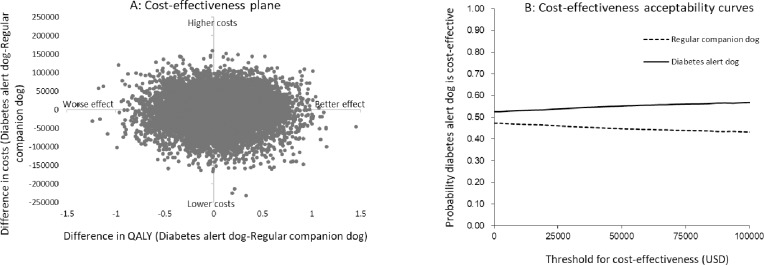
Result of probabilistic analysis for diabetes alert dog owners. Panel A: Cost-effectiveness plane based on 10,000 iterations illustrating the distribution of the ICERs. Panel B: Cost-effectiveness acceptability curves showing the probability that a certified diabetes alert dog is cost effective at different thresholds for cost effectiveness.

The result from the probabilistic sensitivity analysis for diabetes alert dog owners is illustrated in the cost-effective plane in Fig 3 (panel A). The distribution shows that also these results are uncertain. A certified diabetes alert dog is associated with a decrease in costs in 54 percent of the simulations and a gain in QALYs in 57 percent of the simulations. Panel B shows that a certified diabetes alert dog is cost effective in approximately 55 percent of all threshold values.

The reliability of the results was tested in eleven deterministic sensitivity analyses (Tables 5 and 6). None of the sensitivity analyses altered the results for the diabetes alert dogs, compared to the base-case analysis, Table 6. Increasing the costs and decreasing the HRQoL for owners with a retired physical service dog, a physical service dog resulted in incremental cost of approximately 23,000 USD, Table 5. Using a health care perspective when calculating the cost effectiveness of a physical service dog gives an ICER of approximately 24,000 USD.

**Table 5:** Results of sensitivity analyses for physical service dog owners.

Sensitivity analyses				
Scenario		Incremental cost (USD)	Incremental QALY	ICER
1	Discount rate (cost and QALYs): 0%	-7,519	0.32	Dominant
2	Discount rate (cost and QALYs): 5%	-5,105	0.26	Dominant
3	Short life span of the dog‡	-4,730	0.26	Dominant
4	Long life-span of the dogɤ	-6,499	0.30	Dominant
5	Dogs retires at the age of 8	-6,012	0.27	Dominant
6	Dogs retires at the age of 12	-5,926	0.29	Dominant
7	Cost increase and HRQoL decrease for owners with a retired dog*	5,783	0.25	22,763
8	Analysis including related and unrelated costs (all costs)	-22,913	0.28	Dominant
9	Purchasing a fully trained dog (17,569 USD)	-109,766	0.40	Dominant
10	Health-care perspective	6,643	0.28	23,587
11	Societal perspective without productivity losses	-170	0.28	Dominant

*Using an exponential cost increase and exponential HRQoL decrease

‡30 percent of the dogs have died at the age of 11

ɤ 10 percent of the dogs have died at the age of 11

**Table 6:** Results of sensitivity analyses for diabetes alert dog owners.

Sensitivity analyses				
Scenario		Incremental cost (USD)	Incremental QALY	ICER
1	Discount rate (cost and QALYs): 0%	-5,869	0.07	Dominant
2	Discount rate (cost and QALYs): 5%	-3,779	0.05	Dominant
3	Short life span of the dog‡	-3,422	0.05	Dominant
4	Long life-span of the dogɤ	-4,983	0.06	Dominant
5	Dogs retires at the age of 8	-4,649	0.05	Dominant
6	Dogs retires at the age of 12	-4,434	0.06	Dominant
7	Cost increase and HRQoL decrease for owners with a retired dog*	-420	0.00	Dominant
8	Analysis including related and unrelated costs (all costs)	-1,643	0.06	Dominant
9	Purchasing a fully trained dog (17,569 USD)	-21,998	0.08	Dominant
10	Health-care perspective	1,543	0.06	Dominant
11	Societal perspective without productivity losses	-5,209	0.06	Dominant

*Using an exponential cost increase and exponential HRQoL decrease

‡30 percent of the dogs have died at the age of 11

ɤ 10 percent of the dogs have died at the age of 11

## Discussion

There is an ongoing debate regarding means and solutions that can meet the needs of people with a high health-care consumption. With many alternative options and scarce resources, benefits as well as costs for different interventions need to be considered to determine which alternative should get priority. This is essential when a treatment is provided within the public health-care system. Cost-effectiveness analysis is a decision-making tool to help identifying the most beneficial interventions to maximize the value of medical care.

The present study is a first attempt to use health-economic decision modeling to assess the cost-effectiveness of physical service dogs and diabetes alert dogs. The use of companion dog as comparator was chosen to capture the incremental effect of the education of the dog. If the alternative to a certified dog is not having a dog, our comparator design is conservative and most probably underestimate the positive effects. The results indicates that a certified dog is a cost-effective intervention. This is mainly explained by reduced health-care consumption and, consequently, reduced health-care costs. Improvements in HRQoL also contribute to the results. The QALY change is entirely explained by an improvement in the dog owners’ HRQoL. The physical service dog owners have notably low HRQoL, fewer QALYs and high resource use. However, the QALY gain in the physical service dog group is higher than in the diabetes alert dog group, which indicates that the physical service dog owners, on average, have more severe conditions and benefit relatively more from the certified dog.

To our knowledge, no previous studies have examined the cost effectiveness of certified dogs. Nor has anyone attempted to estimate the long-term effects of certified dogs. One strength of this study is the long-term extrapolation of health outcomes and costs. It makes it possible to account for the full effect of a certified dog, both in terms of costs and health outcomes. In an ordinary analytic approach, the long-term effects would not have been captured. However, even though the extrapolation increases the relevance of the results for decision-making, it also introduces more uncertainty in the results.

In the present study, we use data from the “service and hearing dog project”, a project that was performed as an exploratory study with pre-post design, a design associated with methodological weaknesses. The data on health-care utilization was based on self-reports by the participants with a recall period of three months, which may lead to over- or underestimation of resources used, due to recall bias. In addition, the participants had to judge whether the health-care utilization was related or not to the physical impairment that qualify them for the certified dog. This may have been difficult for the participants to decide. Also, the participants in the study were made up of a self-selected sample. This may of course affect the generalizability of the results. The statistical uncertainty in the data, both due to the study design and the heterogeneity of the participants, is confirmed in the probabilistic sensitivity analysis where the ICERs are spread over all four quadrants in the cost-effectiveness plane. It indicates an uncertainty in the decision to implement the strategy. However, in Sweden, decisions on implementation need to consider other aspects than cost effectiveness and uncertainty of results. For example, severity of the condition and implications for the overall health-care budget.

In addition to investigating the statistical uncertainty, a decision model also makes it possible to determine how different assumptions and simplifications affect the result. In a Swedish setting, physical service and diabetes alert dog training can be carried out in three different ways: by the owner in collaboration with a certified instructor, by the owner alone, and by a certified instructor. With the latter alternative, a person can purchase a fully trained dog. The base-case scenario analyzed in this study is that owners train the dog in collaboration with a certified instructor based on the procedure in the “service and hearing dog project”. To analyze if the result differed depending on type of education, a sensitivity analysis of purchasing a fully trained dog was conducted. The analysis showed that type of education did not affect the result, assuming that type of education renders the same results. There is an ongoing discussion regarding which costs to include when conducting an economic evaluation [17]. An analysis including all health-care utilization (related and unrelated) was therefore conducted. However, the inclusion of unrelated costs had no effect on the result. Analyzing other assumption or simplifications such as different discount rates, life span of the dog, the dog’s retirement age etc. did not change the overall results either. Analyzing the cost effectiveness of a physical service dog from a health care perspective had an impact on the cost effectiveness, changing the intervention from being cost-saving to cost 24,000 USD/QALY. When adopting a cost increase in combination with a HRQoL decrease when the dog retired also had an impact on the ICER, it yielded a cost per QALY of approximately 23,000 USD.

This attempt to conduct a health-economic decision analysis is associated with uncertainty. However, complex decisions based on a variety of data will always be uncertain to some extent. The cost-effectiveness acceptability curves showed that the probability of a physical service dog being cost effective increases as the threshold increase and the probability for a diabetes alert dog being cost effective at different thresholds is approximately 55 percent. The analysis is built upon rather conservative assumptions and it has not been possible to account for improvements in the skills of the dog or in the collaboration between the dog and the owner over time. This means that the estimated effects of having a certified dog may be underestimated.

## Conclusions

This study indicates that a certified dog is cost saving in comparison with a regular companion dog for individuals with functional impairments or chronic diseases. The uncertainty of the analysis implies that further studies are needed to confirm these results. Nevertheless, physical service dogs and diabetes alert dogs show potential to be a valuable support, and health-economic analyses are a useful tool to provide this information.

## Supporting information

S1 Appendix Unit costs. (DOCX)

S2 Appendix CHEERS checklist. (PDF)
