# Supplementary material for: Certified service dogs – A cost-effectiveness analysis appraisal
Source: PLoS One. 2019 Sep 12;14(9):e0219911. doi: 10.1371/journal.pone.0219911 (PMC6742471; doi:10.1371/journal.pone.0219911)
Supplement: S1 Appendix — (DOCX) [file pone.0219911.s001.docx]

| Parameter | Cost (USD) | Unit | Reference |
| --- | --- | --- | --- |
| **Health-care costs** | | | |
| Emergency care | 1,250 | Per visit | [1] |
| Ambulance | 378 | Per emergency |  |
| Visit to physicians (hospital) | 396 | Per visit | [2] |
| Visit to physicians (health center) | 257 | Per visit | [1] |
| Home visit physicians | 514 | Per visit | [1, 2] |
| Telephone contact physician | 86 | Per call | [1, 2] |
| Visit to nurse | 159 | Per visit | [2] |
| Home visit nurse | 317 | Per visit | [2] |
| Telephone contact nurse | 53 | Per call | [2] |
| Visit to physiotherapist | 124 | Per visit | [1] |
| Home visit physiotherapist | 249 | Per visit | [1, 2] |
| Visit to occupational therapist | 195 | Per visit | [1] |
| Home visit occupational therapist | 391 | Per visit | [1, 2] |
|  |  |  |  |
| **Municipal services** | | | |
| Home-help services | 54 | Per hour | [2, 3] |
| Personal assistance | 36 | Per hour | [3] |
| Escort/Accompanying person | 36 | Per hour | [3] |
| Transportation service | 42 | Per trip | [4] |
|  |  |  |  |
| **Informal care** | 9 | Per hour | [5] |
| **Sick leave** | 42 | Per hour | [6] |
|  |  |  |  |
| **Dog** | |  |  |
| Purchase dog | 1,515 |  | Patient survey |
| Dog training | 7,746 |  | Patient survey |
| Suitability tests | 193 |  | [7] |
| Yellow cape | 94 |  | [7] |
| Red cape | 117 |  | [7] |
| Annual health declaration | 59 |  | [8] |
| Annual certification maintenance test | 88 |  | [8] |
| Annual costs* | 1,332 |  | Patient survey |
| Purchase a fully trained dog | 17,569 |  | [8] |
| *Annual costs includes costs for food, insurance and veterinary costs. | | | |

**References**

1. Sydöstra Sjukvårdsregionen. Priser och ersättningar för Sydöstra sjukvårdsregionen 2017. Available from: <https://plus.rjl.se/info_files/infosida41089/prislista_2017_slutversion_10_0.pdf>.

2. Sveriges Kommuner och Landsting. Statistik om hälso- och sjukvård samt regional utveckling 2015. Available from: <http://webbutik.skl.se/bilder/artiklar/pdf/7585-337-6.pdf?issuusl=ignore>.

3. Sveriges Kommuner och Landsting. Årets jämförelse av KPB-nyckeltal [Dataset]. Available from: <https://skl.se/ekonomijuridikstatistik/statistik/kostnadperbrukarekpb/aretsjamforelseavkpbnyckeltal.803.html>.

4. Holmstrom A, Danielsson C. Special transport services and national special transport services 2015. Sveriges officiella statistik. 2016.

5. Swedish Statistics (SCB). Average monthly salary, gender and year [Dataset]. Available from: <http://www.statistikdatabasen.scb.se/pxweb/sv/ssd/START__AM__AM0110__AM0110B/LoneSpridSektorYrkA/table/tableViewLayout1/?rxid=7f178888-a00e-4e65-b38d-ca80424de359>.

6. European Commission Eurostat. Hourly labour costs. Available from: <http://ec.europa.eu/eurostat/statistics-explained/index.php/Hourly_labour_costs>.

7. The Swedish Association of Service Dogs. Kostnadskalkyl för utbildning [In Swedish]. Available from: <http://www.soshund.se/kostnadskalkyl-for-ekipage-i-utbildning/>.

8. The Swedish Association of Service Dogs. Personal communication, November 2, 2014.
