# Supplementary material for: Certified service dogs – A cost-effectiveness analysis appraisal
Source: PLoS One. 2019 Sep 12;14(9):e0219911. doi: 10.1371/journal.pone.0219911 (PMC6742471; doi:10.1371/journal.pone.0219911)
Supplement: S2 Appendix — (PDF) [file pone.0219911.s002.PDF]

CJASN
Clinical Journal of American Society of Nephrology

JASN
JOURNAL OF THE AMERICAN SOCIETY OF NEPHROLOGY

Adapted CHEERS checklist for economic evaluation of health interventions

Manuscript ID:

Section/Topic	Checklist Item	Response
**Title/Subtitle**	Study identified as an economic evaluation and describes the interventions compared	Yes
**Background and objectives**	Provides an explicit statement of the broader context for the study explicitly presenting the study question and its relevance for health policy or practice decisions	Yes
**Target Population and Sub-groups**	Describes the characteristics of the base case population and sub-groups analyzed, including why they were chosen	Yes
**Setting and Location**	States relevant aspects of the system(s) in which the decision(s) need(s) to be made	Yes
**Study Perspective**	Describes the perspective of the study and relates this to the costs being evaluated	Yes
**Comparators**	Describes the interventions or strategies compared and states why they were chosen	Yes
**Time Horizon**	States the time horizon(s) over which costs and consequences are being evaluated	Yes
**Discount Rate**	Reports the choice of discount rate(s) used for costs and outcomes and says why appropriate.	Yes
**Choice of Health Outcomes**	Describes what outcomes were used as the measure(s) of benefit in the evaluation and their relevance for the type of analysis performed	Yes
**Measure of Effectiveness**	*Single study-based estimates:* Describes fully the design features of the single effectiveness study, and why the single study was a sufficient source of clinical effectiveness data	Yes
	Synthesis-based estimates: Describes fully the methods used for identification of included studies and synthesis of clinical effectiveness data	N/A
**Measurement and valuation of preference based outcomes**	Describes the population and methods used to elicit preferences for outcomes	Yes
**Estimating resources and costs**	*Single study-based economic evaluation:* Describes approaches used to estimate resource use associated with the alternative Interventions, primary or secondary research methods for valuing each resource item in terms of its unit cost, and any adjustments made to approximate to opportunity costs	N/A
	Model-based economic evaluation: Describes approaches and data sources used to estimate resource use associated with model health states. Describes primary or secondary research methods for valuing each resource item in terms of its unit cost and any adjustments made to approximate to opportunity costs	Yes
**Currency, price date, and conversion**	Reports the dates of the estimated resource quantities and unit costs, methods for adjusting estimated unit costs to the year of reported costs if necessary, and methods for converting costs into a common currency base and the exchange rate	Yes
**Choice of model**	Describes and gives reasons for the specific type of decision analytical model used	Yes
**Assumptions**	Describes all structural or other assumptions underpinning the decision-analytical model	Yes
**Analytical methods**	Describes all analytical methods supporting the evaluation. This could include methods for dealing with skewed, missing, or censored data; extrapolation methods; methods for pooling data; approaches to validate or make adjustments (such as half cycle corrections) to a model; and methods for handling population heterogeneity and uncertainty	Yes
**Study parameters**	Reports the values, ranges, references, and, if used, probability distributions for all parameters, reasons or sources for distributions used to represent uncertainty where appropriate.	Yes
**Incremental costs and outcomes**	For each intervention, reports mean values for the main categories of estimated costs and outcomes of interest, as well as mean differences between the comparator groups. If applicable, reports incremental cost-effectiveness ratios.	Yes
**Characterizing uncertainty**	*Single study-based economic evaluation:* Describes the effects of sampling uncertainty for the estimated incremental cost and incremental effectiveness parameters, together with the impact	N/A
	Model-based economic evaluation: Describes the effects on the results of uncertainty for all input parameters, and uncertainty related to the structure of the model and assumptions	Yes
**Characterizing heterogeneity**	If applicable, reports differences in costs, outcomes, or cost-effectiveness that can be explained by variations between subgroups of patients with different baseline characteristics or other observed variability in effects that are not reducible by more information	Yes
**Discussion**	Summarizes the key findings and describes how they support the conclusions reached	Yes
	Discusses the limitations and the generalizability of the findings and how they fit with current knowledge	Yes

Source: Husereau D, Drummond M, Petrou S, Carswell C, Moher D, Greenberg D, Augustovski F, Briggs AH, Mauskopf J, Loder E. Consolidated Health Economic Evaluation Reporting Standards (CHEERS) statement. BMJ 2013; 346: f1049

*This checklist has been adapted by CJASN and JASN for author and editor usage.

December 2017
